# Reply to Moreno et al. Comment on “Sallam, M. ChatGPT Utility in Healthcare Education, Research, and Practice: Systematic Review on the Promising Perspectives and Valid Concerns. *Healthcare* 2023, *11*, 887”

**DOI:** 10.3390/healthcare11222955

**Published:** 2023-11-13

**Authors:** Malik Sallam

**Affiliations:** 1 Department of Pathology, Microbiology and Forensic Medicine, School of Medicine, The University of Jordan, Amman 11942, Jordan; malik.sallam@ju.edu.jo; 2 Department of Clinical Laboratories and Forensic Medicine, Jordan University Hospital, Amman 11942, Jordan

I would like to thank the authors for their commentary on the publication “ChatGPT Utility in Healthcare Education, Research, and Practice: Systematic Review on the Promising Perspectives and Valid Concerns” [1,2]. I value this opportunity to address the points raised in the commentary, and to engage in a communication that can hopefully enrich the collective understanding of the role of artificial intelligence (AI) in healthcare education, research, and practice. This aim was one of the initial motivations of the review in light of the ongoing debate surrounding AI’s benefits and risks and its potential impact on the academic landscape, with a special focus on the healthcare domain given the widespread use of ChatGPT [3,4,5].

As highlighted in the review and the commentary, the significance of AI, particularly large language models (LLMs) like ChatGPT, in healthcare education, research, and practice cannot be understated [1]. Therefore, the growing importance of addressing the implications and challenges associated with implementing AI in healthcare should be properly acknowledged [6]. The commentary’s recognition of the review’s attempt to synthesize the benefits and limitations of ChatGPT demonstrated a shared commitment to reaching a comprehensive understanding of its promising perspectives and valid concerns.

Regarding the concerns raised in the commentary, I fully acknowledge the potential limitations of the review, which stem from the methodology used for the systematic search and the inclusion of various types of research records (e.g., preprints, editorials, communications, etc.), as well as from the evolving nature of the research subject with subsequent inherent challenge in accurate assessment of the long-term impact of LLMs. Additionally, I would like to highlight that the necessity for further elaboration on the review limitations was raised and addressed during the peer review process, including the issues of single authorship, absence of quality assessment of the included records, and the rapid evolution of the research subject. These issues were missing from the initial preprint and were included in the final published form of the review [1,7]. Furthermore, the review clearly stated that the results should be carefully interpreted in light of the inherent limitations [1]. Thus, the conclusions of the review were drawn from the available data at the time and were subject to the dynamics of the rapidly advancing research subject, as stated in the original review [1].

While I recognize the essential role of post-publication scholarly critique, I firmly believe that such exchanges should adhere to the highest standards of accuracy and respect for context [8]. In light of this, I would like to address specific points within the commentary that I consider to be addressed within the original publication as follows:

1. While I hold the PROSPERO registration in high regard as a valuable component of systematic reviews, it is pertinent to acknowledge that the nature of the review did not involve human subjects, thus rendering PROSPERO registration non-mandatory in this particular context [9,10]. Additionally, it is important to emphasize that the preprint version of the manuscript was accessible for more than a month prior to the formal acceptance of the publication following editorial evaluation and multiple rounds of peer review [7]. Furthermore, it is important to highlight that rigorous adherence to the established methodologies was followed in the review, with commitment to transparent reporting standards, as meticulously explained in the Methods section of the published manuscript [1].
2. Contrary to the commentary’s claim, the search strategy employed was meticulously outlined in the published manuscript [1]. The specific search term, databases utilized, and outcomes were elucidated in the Methods section of the manuscript. The selection process, tailored to the review’s broader objectives, was executed thoroughly, in line with the review objectives [1]. The decision to use a single keyword was based on the specificity of the review topic, which focused on “ChatGPT” in the context of healthcare practice, healthcare education, and academic writing. At the time of the search, which concluded on 16 February 2023, “ChatGPT” was a relatively new and emerging concept in the scientific research field. For example, this term first appeared in the PubMed database in November 2022 (https://pubmed.ncbi.nlm.nih.gov/?term=chatgpt&size=200, accessed on 19 October 2023). Given this context, a focused search term would yield the most relevant and comprehensive results for the specific review objectives in line with the PRISMA guidelines. The comprehensiveness of the review was also the basis for the inclusion of various types of content (original papers, preprints, reviews, commentaries, etc.). Additionally, the use of Google Scholar besides PubMed, given its rapid comprehensive indexing of a wide range of scholarly and non-scholarly literature, ensured that the likelihood of missing relevant references would be negligible [11].
3. The issue of missing a priori qualitative analysis methodology in the review should be interpreted in light of the review’s focus on ChatGPT’s implications rather than methodological intricacies. Thus, a broad categorization of ChatGPT’s implications in healthcare was considered in order not to miss important peculiar observations of ChatGPT’s benefits/concerns in the included records. Thus, it is important to contextualize this consideration within the specific objectives of the review.
4. Two important points that appear to be overlooked in the commentary are related to the publication comprising me as a single author and the absence of quality assessment of the included records. These issues were addressed during the peer review process and were also mentioned explicitly in the limitations sub-section of the publication, as mentioned earlier in this letter [1].

Nevertheless, I have to re-emphasize that I truly acknowledge the role of scholarly collaboration, which undoubtedly enriches research, and this will be considered in my future work.

Finally, I have to acknowledge that it is my sincere belief that academic discourse thrives on constructive engagement. Therefore, I would like to thank the authors of the commentary for their notable level of interest in the review, which signifies the scholarly relevance and potential real-world implications of its findings. 
